# George Huntington: a legacy of inquiry, empathy and hope

**DOI:** 10.1093/brain/aww165

**Published:** 2016-07-14

**Authors:** Alice Wexler, Edward J. Wild, Sarah J. Tabrizi

**Affiliations:** aww165-1 1 Hereditary Disease Foundation, 3960 Broadway, 6th Floor, New York, New York 10032, USA; aww165-2 2 Huntington’s Disease Centre, UCL Institute of Neurology, National Hospital for Neurology and Neurosurgery, Queen Square, London WC1N 3BG, UK

## Abstract

On the centenary of George Huntington's death, Wexler *et.al.* reconsider the setting and the collaborative effort that produced his description of “hereditary chorea,” today Huntington's disease. Tracing the changing identity of this illness, they discuss the legacy of eugenics, the search for the gene, and ongoing research toward a cure.

In the 100 years since the death of George Huntington in 1916, the disorder he described as a ‘medical curiosity’ has become a focus of intense medical and scientific interest, in part because of the contribution of families in generating knowledge about this family disease. As many writers have noted, George Huntington’s own family played a crucial role in defining this illness (Harper, 2002). What has been less appreciated is that the affected families he described also played a role, in ways that George Huntington himself acknowledged. Not quite 22 years old (Fig. 1), just graduated from the College of Physicians and Surgeons in New York City, and with little clinical experience, no established medical practice, and no patients of his own with the disorder, he wrote an account in 1872 that William Osler considered one of the most succinct and accurate portraits of a disease ever written (Osler, 1893). It was not the earliest medical account of hereditary chorea but it was certainly the most complete. And for social and cultural as well as medical and scientific reasons, it played a far more important role in defining the discrete clinical entity that soon came to be known as ‘Huntington’s chorea’ and by the late 1960s, as ‘Huntington’s disease’. Despite considerable recognition during his lifetime, George Huntington remained a small town family physician but not a provincial or isolated one. He was aware that his paper had drawn the attention of the medical profession at home and abroad and that this had helped reveal the disease in many parts of the world. He was in touch with some eminent clinicians of his day, including Osler, and an invited speaker on Huntington’s chorea at medical societies such as the influential New York Neurological Society. At a time when medicine was becoming increasingly ‘scientific’, he too placed his hope in research, although he chose not to pursue research himself. Alluding to the unknown pathology of chorea, which had intrigued him from the start, he trusted ‘that science, which has accomplished such wonders through the never-tiring devotion of its votaries, may yet “overturn and overturn, and overturn it,” until it is laid open to the light of day’. (Huntington, 1872).

**Figure 1:**
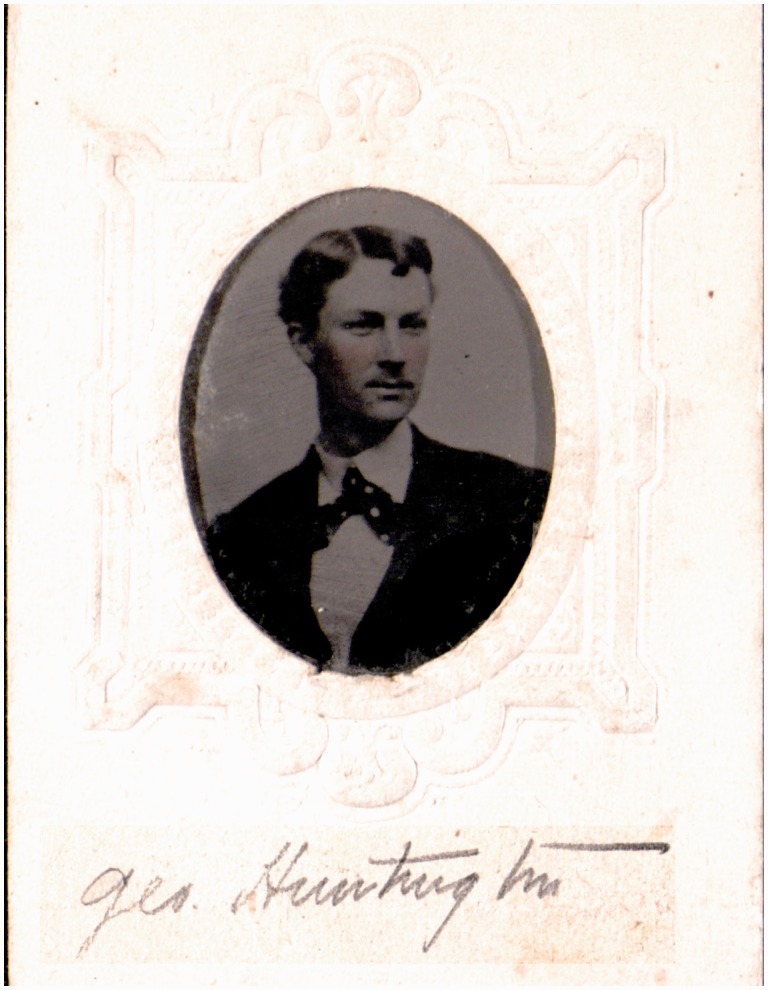
**George Huntington *c.* 1868.** (Mulfold Album, Courtesy of the East Hampton Library, Pennypacker Long Island Collection). A naturalist as well as a hunter despite his severe asthma, George Huntington also sketched and painted landscapes and local scenes all his life, a practice that no doubt contributed to his keen powers of observation. His travel reports, published in a Long Island newspaper, testify to his early skill as a writer.

## A tale of two families

**Figure 2:**
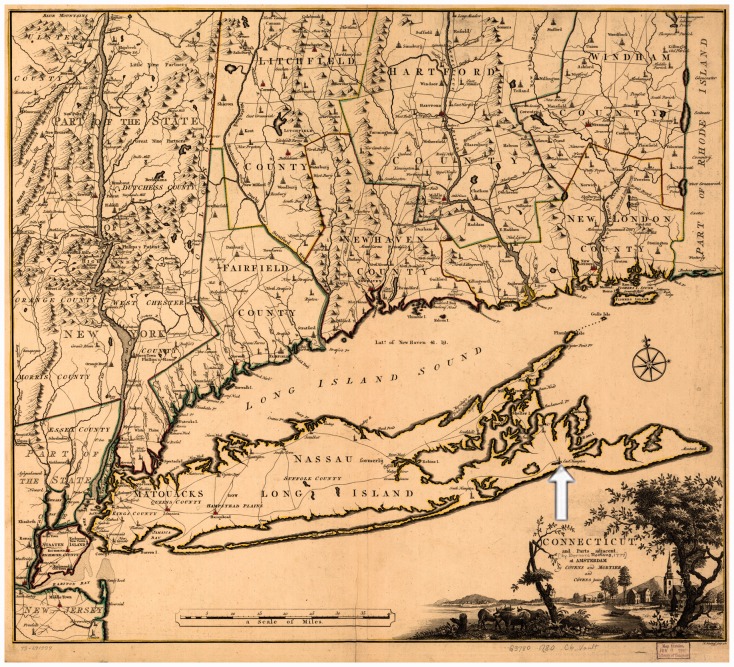
**Map of Connecticut and environs, *c*. 1780.** Including East Hampton (white arrow) [H. Klockhoff and B. Romans, ‘Connecticut and Parts Adjacent‘ (Amsterdam: Covens and Mortier and Covens Jur., 1780) Library of Congress, Geography and Map Division].

On 12 June 1806, in the long-established farming and fishing village of East Hampton, at the far eastern end of Long Island, about 60 miles northeast of New York City, USA (Fig. 2), Captain David Hedges, a member of the local gentry, awoke to find his wife missing. A search ‘thro fields of grain to the shore’ did not find her ‘and there is every reason to believe she has precipitated herself into the surf’, reported the local newspaper. ‘Mrs. Hedges was about 40 years of age, and was much esteemed by her neighbors’ the obituary continued. ‘This extraordinary step is attributed to her extreme dread of the disorder called St. Vitus’ dance, with which she began to be affected, and which her mother now has to a great degree. From some arrangements of her clothing it appears she had for some time contemplated her melancholy end.’ (*The Suffolk Gazette,* 30 June 1806).

Phebe Hedges and her mother, also called Phebe, descended from one of the oldest and most prominent East Hampton families who had settled the town in the 17th century from New England. [They bore no known relation to the East Anglia families whom P. R. Vessie and M. Critchley later and erroneously characterized as disreputable ancestors of many Huntington’s families in the USA (Wexler, 2008)]. They were patients of George Huntington’s grandfather, known as the Honourable Abel Huntington (1777–1858), a highly respected physician who had arrived in East Hampton from Connecticut in 1797 and found the disease well established there. The Huntingtons were long considered relative newcomers but they quickly won the esteem of their neighbours and played active roles in the local community, state, and even nationally. When Abel’s political activities drew him away from East Hampton, his physician son, George Lee Huntington (1811–81), became the doctor to the descendants of Phebe and Captain David Hedges. George Huntington, the son of George Lee and his wife Mary, born in 1850 and educated in East Hampton, was thus a third generation physician in a medical family that had lived alongside families with hereditary chorea since the end of the 18th century and was well situated to observe, over multiple generations, who did and did not have the disease.

## ‘A medical curiosity’

In the fall of 1871, soon after graduation from medical school, George Huntington moved to Pomeroy, Ohio. His cousin had married a clergyman in Pomeroy and suggested the young George open a medical practice there. Invited to present a paper to the local Meigs and Middleport Academy of Medicine in nearby Middleport, he chose to talk ‘On Chorea’, perhaps because he had recently observed cases of childhood (Sydenham’s) chorea in the clinic as a medical student in New York City and was struck by the differences from the chorea he had observed back home. Most of the paper, in fact, described this common type of chorea but in the final paragraphs George Huntington outlined three predominant characteristics of a type of chorea he believed to be present ‘exclusively on the east end of Long Island’. First was its hereditary nature, for it was ‘confined to certain and fortunately a *few* families, and has been transmitted to them, an heirloom from generations away back in the dim past’. It differed, however, ‘from the general laws of so-called hereditary diseases’ in which the disease may skip a generation. ‘Unstable and whimsical as the disease may be in *other* respects, in *this* it is firm, it never skips a generation to again manifest itself in another; once having yielded its claims, it never regains them.’

Second, was the tendency to what George Huntington called, in 19th century parlance, ‘insanity, and sometimes that form of insanity which leads to suicide’. (19th century meanings of ‘insanity’ ranged from disturbances of mood to disorders of thought and behaviour as well as personality changes. Suicide typically cast the deceased as having suffered from insanity.) He also noted specifically the tendency to cognitive impairment. ‘As the disease progresses the mind becomes more or less impaired, in many amounting to insanity, while in others mind and body both gradually fail until death relieves them of their sufferings’. He further described a lack of self-awareness and loss of inhibitions on the part of sufferers, offering a far more nuanced description than had been given in earlier accounts.

Finally, he noted that the symptoms generally manifested in adult life, most often between 30 and 40 years of age, increasing very gradually ‘until every muscle in the body becomes affected (excepting the involuntary ones)’. The disease progressed inexorably without any periods of remission. ‘When once it begins it clings to the bitter end.’ Huntington ended by acknowledging that he knew nothing of its pathology and offered his account, ‘not that I considered it of any great practical importance to you, but merely as a medical curiosity, and as such it may have some interest’.

**Figure 3:**
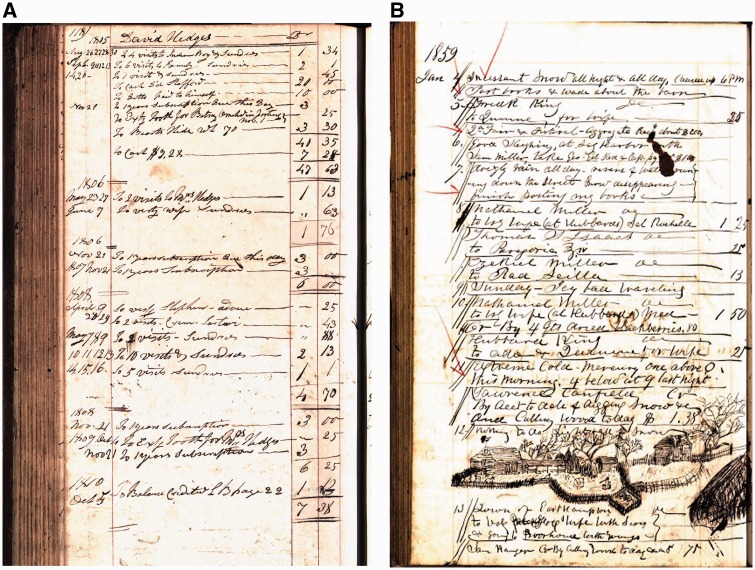
**Pages from the ledgers and daybooks of George Huntington’s grandfather Abel (**A**) and father George Lee (**B**) who cared for the families whose illness George later described (Courtesy of the East Hampton Library, Pennypacker Long Island Collection).** See Supplementary material for further references.

It is sometimes assumed that the East Hampton families with chorea were George Huntington’s patients. In fact, George himself never established a medical practice in East Hampton, although he tried to briefly in the summer of 1872. The cases he described were patients of his father and grandfather (Fig. 3) and he always acknowledged his debt to them, indicating that without their facts and observations his paper could not have been written (Osler, 1893). (The Huntington household, in fact, included a number of educated, acute observers whose intimate local knowledge was shared with the young George—apart from his parents, there was George’s aunt, the poet and novelist Cornelia Huntington who published a novel filled with allusions to ‘the sins of the fathers’ and with a ‘nervous’ central character; also their long-time boarder John Wallace, an educated Scottish churchman who founded the local Episcopal church.)

But he also incorporated the knowledge of the East Hampton families themselves, including their recognition that this malady was hereditary. Actually many ailments were considered to be inherited in the 19th century. It is not surprising that, in an old community with memory going back many generations, the affected families—indeed the entire community—recognized that this disorder was hereditary. Nor is it surprising that they theorized about its genealogical origins and even spoke of families ‘who belonged to the disease’. What surprises is that they were expert diagnosticians who had precise ideas of who did or did not have this illness in each generation. They understood its terrors, for as George Huntington wrote, ‘it is spoken of by those in whose veins the seeds of the disease are known to exist, with a kind of horror, and not at all alluded to except through dire necessity, when it is mentioned as “*that disorder*”’. (According to Henry P. Hedges, a distant relative of Phebe Hedges and a historian of East Hampton, it was also called St. Vitus’s dance although ‘the subject is avoided by most people as distasteful’.) The families also understood its trajectory, for ‘its end is so well known to the sufferer and his friends, that medical advice is seldom sought’. Years after he published ‘On Chorea’, George Huntington the artist as well as the physician beautifully captured both his own childhood awe on first encountering persons with chorea and his empathy as an adult for the suffering of those who had been his family’s neighbours and friends:

‘Driving with my father through a wooded road leading from East Hampton to Amagansett, we suddenly came upon two women, mother and daughter, both tall, thin, almost cadaverous, both bowing, twisting, grimacing. I stared in wonderment, almost in fear. What could it mean? My father paused to speak with them, and we passed on. Then my Gamaliel like instruction began; my medical education had its inception. From this point my interest in the disease has never wholly ceased. Then came the hanging of D.H. in his blacksmith shop. He was a victim of incipient chorea, knew it, possibly had been waiting for it, the “Sanctus Invictus,” and well knowing the character of the foe he must meet, the so dreaded, the long expected, the conquering, he cut short the taper and his life went out. Other victims had sought the same refuge again and again by drowning. Others, of a different nervous organization perhaps, lived on if not content, still seemingly reconciled to Fate, until mind and body both exhausted they fell asleep.’

## Huntington’s chorea in the crosshairs of psychiatry, neurology, genetics and eugenics

**Figure 4:**
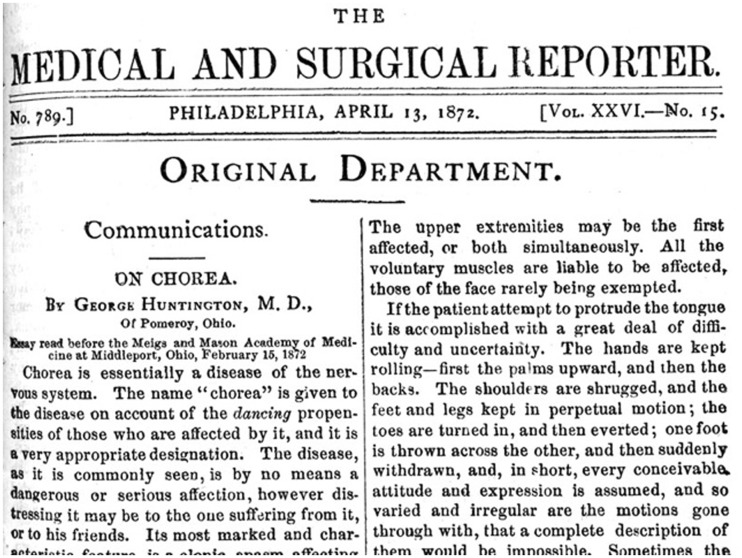
**George Huntington’s 1872 paper ‘On chorea’ in the *Medical and Surgical Reporter*.** His later talks before various medical societies on Huntington’s chorea were published, in the *Brooklyn Medical Journal* (1895), the *Transactions of the Tri-State Medical Association* (1903), and *The Journal of Nervous and Mental Disease* (1910). His unpublished papers are in the Special Collections of the Columbia University Health Sciences Library, New York City, and the Pennypacker Long Island Collection, East Hampton Library, New York.

Although George Huntington’s paper was immediately published in a reputable Philadelphia medical journal, *The Medical and Surgical Reporter* (Fig. 4), it attracted little notice at first apart from a brief abstract in Virchow and Hirsch’s *Jahresbericht* or Yearbook of Important Medical Writings for the Year 1872. Two years later, the eminent Italian neuropathologist Camillo Golgi footnoted it in a paper noting dramatic changes in the cortex and striatum of a deceased 42-year-old male patient with chorea, mental disturbance, and an ‘hysteric’ mother (Golgi, 1874). However, with the expansion of neurology as a medical specialty in the 1870s and 1880s and the growing population of patients in psychiatric hospitals and asylums in the USA and Europe, clinicians began reporting similar cases with increasing frequency. In 1892 some authors were even claiming that the literature on this disorder was ‘copious’ and that its clinical history was ‘very thoroughly known’. (Osler, 1893; Wexler, 2008). (A few clinicians, including Charcot, considered ‘hereditary chorea’ a variant of the childhood disorder. But by the 1890s most viewed it as distinct.) Ironically, while neurologists claimed Huntington’s chorea as a neurological disease *par excellence,* many more psychiatrists than neurologists actually encountered such patients, typically in the mushrooming population of psychiatric institutions. Those who did were impressed by the contrast this disorder presented with Sydenham’s chorea, including the late onset, the inexorable progression, and the fact that ‘heredity is one of the most remarkable features’. Nonetheless, the names used at the time highlighted features other than heredity, for example chorea of the aged, choreic dementia, dementia choreica, and Osler’s preference, chronic progressive chorea, although soon Huntington’s chorea displaced them all (Wexler, 2008).

With the rediscovery of Mendel’s laws in 1900 and also the establishment of eugenics societies in many countries, the hereditary dimension of Huntington’s chorea—and the specific pattern of inheritance—began to draw increased attention, not only from clinicians but also from biologists studying Mendelian heredity more broadly. In addition to its interest as a case study in human Mendelian inheritance, Huntington’s chorea now appeared to some influential supporters of eugenics as a cause for public health alarm. With the resources of the Carnegie-funded Eugenics Record Office at Cold Spring Harbor, New York, the biologist and eugenics leader Charles B. Davenport in 1911 initiated the first large-scale pedigree study of families with Huntington’s chorea, creating pedigrees that included some 962 alleged cases, living and deceased, in a total of ∼4370 individuals in the northeastern USA. Davenport and Muncey’s 1916 paper ‘Huntington’s Chorea in Relation to Heredity and Eugenics’, was widely cited and largely unchallenged for decades despite Davenport’s extreme eugenic views, which helped to normalize the notion of sterilization as the recommended method of prevention, a notion that prevailed well into the 1960s (Davenport and Muncey, 1916). At that time Hans and Gilmore and later Wexler demonstrated that these pedigrees were riddled with unreliable or clearly erroneous diagnoses and gross genealogical errors, including errors that laid the foundation for subsequent claims about Huntington’s and witches, ‘criminality’, and violence (Hans and Gilmore, 1969; Wexler, 2008). Such claims, repeated even today, deepened the stigmatization of Huntington’s families while discouraging research toward effective treatment. They also gave families further incentives for maintaining secrecy and silence about this disease (Wexler and Rawlins, 2005).

## Scientific advance and social change

Reports on the neuropathology of chorea in adults appeared as early as the 1870s, with researchers generally agreed that the basic lesion was located in the basal ganglia, with the striatum, particularly the caudate nucleus, showing the greatest degree of atrophy. However, there was little agreement on its cause and relatively little progress for decades. The confluence of several developments in the 1960s radically transformed this bleak research landscape. First, the discovery of L-DOPA and its benefits for patients with Parkinson’s disease spurred an international gathering of neurologists in 1967 to organize a Research Group on Huntington’s Chorea. Second, the rise of social movements in the 1960s challenged the legacy of eugenics and encouraged members of families with Huntington’s to become active on their own behalf. Activists such as the North Americans Marjorie Guthrie, widow of the songwriter and singer Woody Guthrie who died with Huntington’s in 1967, and Milton and Nancy Wexler, husband and daughter of recently diagnosed Leonore Wexler, along with Ralph Walker in Canada, Mauveen Jones in the UK, Gerrit Dommerholt in the Netherlands, and Huntington’s family members elsewhere spearheaded efforts to improve care as well as to interest scientists in research. In the late 1960s and 1970s they formed disease advocacy associations in many countries (called self-support organizations or health voluntary agencies) which, along with revolutionary advances in molecular genetics and neuroscience, expanded biomedical interest in Huntington’s. New technologies of gene mapping opened up possibilities for identifying—and perhaps disabling—the aberrant gene. New modes of imaging offered possibilities for understanding—and potentially intervening in—the sequence of pathological changes in the brain.

At a 1972 Centennial Symposium on Huntington’s disease, in Columbus Ohio, near the town where George Huntington presented his landmark paper, some 136 researchers and a few members of Huntington’s families from around the world gathered to commemorate his contribution and develop new directions for research. Out of this meeting came the impetus for a bold collaborative project focused on a unique cluster of affected families in Venezuela who had been diagnosed back in the 1950s by a local physician, Americo Negrette, author of the first monograph ever published on Huntington’s disease (Negrette, 1963). Drawing on the new somatic cell genetics for mapping genes, this project, led by Nancy Wexler, culminated in 1983 in the identification of a genetic marker for Huntington’s (Gusella *et al.*, 1983). Besides demonstrating that the new technology could be used for mapping the human genome, the landmark marker discovery for the first time enabled those at 50% risk for Huntington’s disease to learn either that they were forever free of the disease and were not in danger of passing it on to their children or grandchildren, or that they did carry the abnormal version of the gene and would therefore develop symptoms if they lived long enough: a momentous form of new knowledge. It also inspired the formation of the legendary Huntington’s Disease Collaborative Research Group, again under the leadership of Nancy Wexler and the Hereditary Disease Foundation. After 10 agonizing years of work, the group collectively announced the identification of the abnormal Huntington’s disease gene (Huntington’s Disease Collaborative Research Group, 1993; Wexler, 2012) (Fig. 6).

## Towards a third age of Huntington’s disease

In a coding region of chromosome 4 initially labelled IT-15 (‘interesting transcript 15’), the Collaborative Research Group had found an expanded CAG triplet repeat that segregated completely with Huntington’s disease cases. The gene was soon renamed *HTT* and its protein product baptized ‘huntingtin’. So began a new age of Huntington’s disease: armed now with certainty of the cause of every past, present and future case of Huntington’s disease, researchers could focus their efforts on understanding the gene, studying the protein in its wild-type and mutant forms, elucidating the mechanisms through which mutant huntingtin (mHTT) causes disease, and working on therapies targeting the mutation and its known effects.

Hopes that the gene discovery would lead overnight to a cure for Huntington’s disease quickly sublimated into a realization that a long road lay ahead—but at least, a road illuminated by the certitude conferred by the genetic basis of Huntington’s disease. At every branch-point, paths unconnected to the expanded *HTT* gene could be discounted. This certainty offers researchers on Huntington’s a potent advantage over those studying more common neurodegenerative diseases, which remain almost entirely idiopathic. Our understanding of the complex roles of the protein and the multiple toxicities of its mutant counterpart rapidly burgeoned, but there was solace to be drawn from knowing each was a potential new target for therapeutic development (reviewed in Wild and Tabrizi, 2014; Bate *et al.*, 2015).

The first mouse model of Huntington’s disease, the R6/2, expressing *HTT* exon 1 under control of the human promoter, followed rapidly in 1996 and remains a mainstay in the field thanks to its robust phenotype and rapid progression. mHTT-containing protein aggregates were soon found in its neurons and in the brains of human patients. Numerous model systems from cells to primates and everything in between are now available to elucidate aspects of pathology and explore therapeutics (reviewed in Bates *et al.*, 2015).

**Figure 5:**
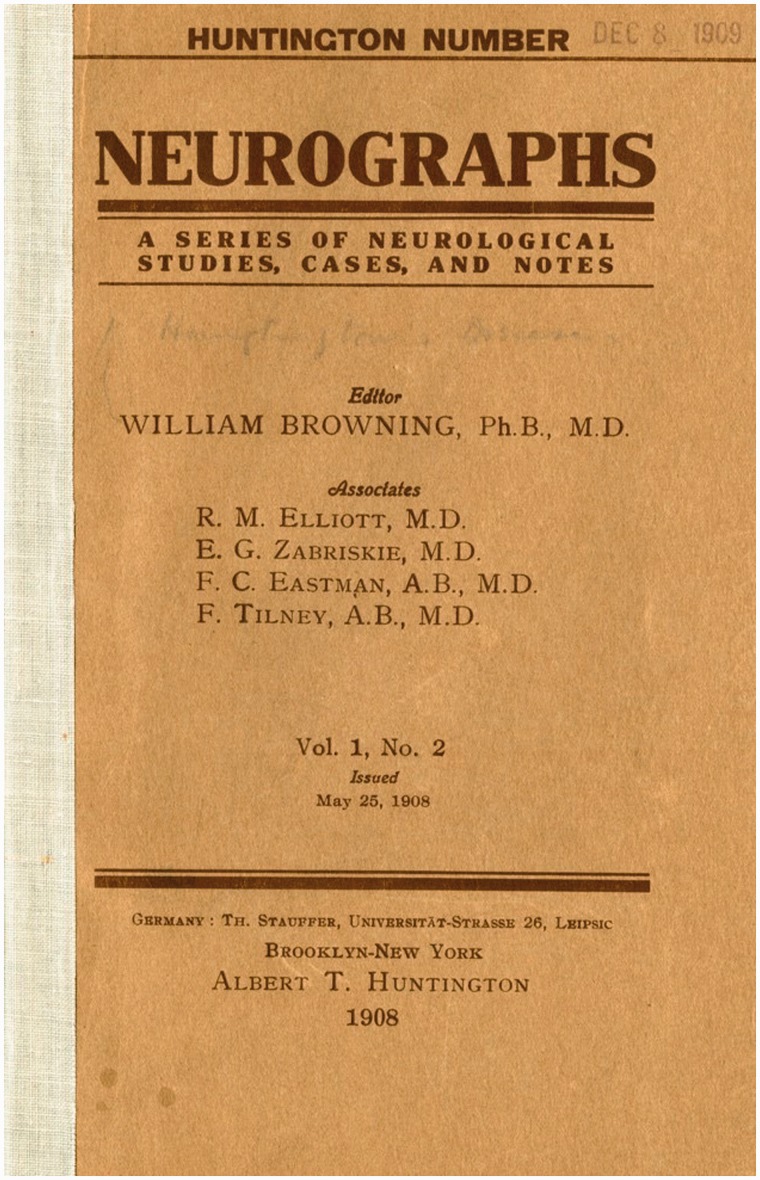
**Huntington special issue of Neurographs.** In 1908 the New York neurologist William Browning devoted an entire issue of his journal *Neurographs* to Huntington’s chorea, emphasizing what he called ‘the world-wide interest’ in the disease.

The first glimmer of tractability came in 2000 from an inducible mouse model in which Yamamoto and colleagues showed that stopping production of the mutant protein after the onset of disability resulted not in the expected slowing of decline but in a reversal of symptoms and neuropathology (Yamamoto *et al.*, 2000). Numerous therapeutic successes followed in mouse models using small-molecule therapeutics targeting an increasing array of rational cellular targets (Fig. 5). Many agents have gone on to be tested in human patients, unfortunately with no success in reversing, delaying or slowing the progression of Huntington’s disease so far: one patient sardonically reflected to one of us, ‘what we really need is a pill to turn humans into mice’. So far, most compounds tested in patients have been ‘neutraceuticals’ or repurposed drugs from other indications which may in part explain their poor performance in the complex context of Huntington’s disease in the human brain (Bates *et al.*, 2015). But things are changing.

**Figure 6:**
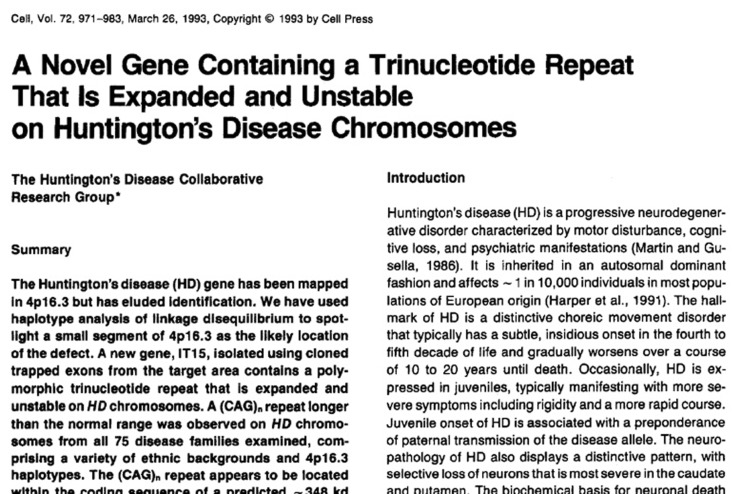
**Collectively authored paper describing the identification of the CAG triplet repeat expansion in IT15 responsible for Huntington’s disease.** This discovery was considered so momentous that it appeared on the front page of *The New York Times* (24 March 1993).

**Figure 7:**
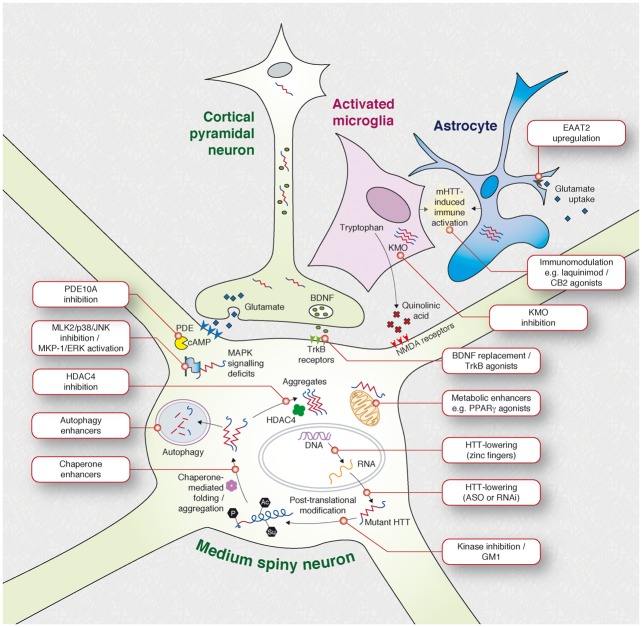
**Overview of current therapeutic targets for Huntington’s disease.** From Wild and Tabrizi (2014).

If we could achieve in humans, using a targeted drug molecule, what was seen in Yamamoto’s conditional *HTT* knockout mouse, we would theoretically treat every facet of Huntington’s disease. Work on such ‘gene silencing’ or ‘huntingtin lowering’ approaches began in Huntington’s disease model animals in 2005 using RNA interference. Similar approaches using antisense oligonucleotides (ASOs)—modified single-stranded DNA—began around the same time. Each aims to reduce HTT expression by targeting its mRNA transcript for removal by the cellular mRNA degradation machinery. For the past decade these technologies have been honed and put through their paces in animal models (Wild and Tabrizi, 2014).

Huntington was almost 22 when he presented his seminal work, and by coincidence it was 22 years after the discovery of the *HTT* gene that the first injection of an ASO therapeutic targeting huntingtin production in the brain was administered into the spinal fluid of a patient with Huntington’s disease in September 2015 in either Vancouver or London (the ongoing safety trial remains double-blinded). Whatever the outcome of this first tentative step, the ‘third age’ of Huntington’s disease—the age of rationally-developed therapeutics targeting the cause and known pathobiology of Huntington’s disease—is now at hand. Crucially, the ASO trial and its successors are empowered by biomarkers and outcome measures established and validated by thousands of Huntington’s disease family volunteers who are giving their time and energy to observational cohort studies knowing they are not likely to benefit themselves but that many others will.

## Conclusion

One hundred years after George Huntington’s death, his eponymous disease is no longer the obscure and shameful malady that it was during his lifetime. Nor is it considered so rare. New studies have shown a much higher prevalence rate, at least in the UK, than was previously reported, about 12 per 100 000 population, although the incidence has remained unchanged (Evans *et al.*, 2013). And while Huntington’s is still incurable, it is no longer untreatable. Medications can reduce choreic movements and ease psychiatric symptoms. Social and psychological support can do much to improve quality of life, for unaffected family members as well as for those at risk and those living with the disease. New technologies such as *in vitro* fertilization and preimplantation genetic diagnosis (PGD) offer increased options for those at risk who wish to have families without passing on the malady (Bates *et al.*, 2015). The activism and imagination of Huntington’s family members, especially the young, has helped reduce the isolation and social stigma that has long added to the suffering associated with the disease. But all these advances are distributed unequally; many people in Huntington’s families, not only in poor countries but also in wealthy ones, do not have access to these benefits. While it is essential that we continue to invest in research on disease-modifying therapies, it is also vital that existing interventions, therapies and services be made accessible to those who need them if the promise of science is truly to be realized.

## Funding

E.J.W is funded by the Medical Research Council. This work was supported in part by the National Institute for Health Research, University College London Hospitals Biomedical Research Centre and the UCL Leonard Wolfson Experimental Neurology Centre.

## Supplementary material

Supplementary material is available at *Brain* online.

## Supplementary Material

Supplementary Data
