# Supplementary material for: George Huntington: a legacy of inquiry, empathy and hope
Source: Brain. 2016 Jul 14;139(8):2326–33. doi: 10.1093/brain/aww165 (PMC4958906; doi:10.1093/brain/aww165)
Supplement: Supplementary Data [file aww165_supp.zip › aww165_Supplementary_Data/brain-2016-00562-File010.pdf]

Some English Language Sources for George Huntington

The unpublished daybooks, ledgers, manuscripts, notes, photographs, drawings, and other materials of George Huntington are in the George Huntington Papers, Archives and Special Collections, Columbia University Health Sciences Library, New York, and the Pennypacker Long Island Collection, East Hampton Library, East Hampton, New York.

By George Huntington:

Huntington G. On Chorea. Medical and Surgical Reporter 1872; 26:317-321.

Huntington G. From our Correspondents (Letter). The Sag Harbor Express March 7, 1872:1.

Huntington G. A Letter from Pomeroy (Letter). The Sag Harbor Express April 4, 1872: 1.

Huntington G. Huntington's Chorea. Brooklyn Medical Journal 1895; 9: 173-174.

Huntington G. Chronic Progressive or Huntington's Chorea. Trans Tri-State Med Assoc 1903; 5:180-185.

Huntington G. Excerpts of paper read before the New York Neurological Society Dec. 7, 1909. Typescript. George Huntington Papers, Archives and Special Collections, Columbia University Health Sciences Library, Box 5, Folder 4.

Huntington G. Recollections of Huntington's Chorea as I Saw It at East Hampton, Long Island, During my Boyhood. Journal of Nervous and Mental Disease 1910; 37: 255-257.

About George Huntington

DeJong RN. George Huntington and his Relationship to the Earlier Descriptions of Chronic Hereditary Chorea. Annals of Medical History 1937; 19:201-210.

Durbach N, Hayden MR. George Huntington: the man behind the eponym. Journal of Medical Genetics 1993; 30: 406-409.

Lanska DJ. Historical Vignette: George Huntington and Hereditary Chorea. Journal of Child Psychiatry 1995; 10: 46-48.

Lanska DJ. George Huntington (1850-1916) and Hereditary Chorea. Journal of the History of the Neurosciences 2000; 9:76-89.

Stevenson CS. "A Biography of George Huntington M.D." Bulletin of the Institute of the History of Medicine 1934; 2:53-76.

Winfield JM. A Biographical Sketch of George Huntington, M.D. Neurographs 1908; 1:89-94.
